# TAK1 mRNA Expression in the Tumor Tissue of Locally Advanced Head and Neck Cancer Patients

**Published:** 2008-02-14

**Authors:** Beatriz Honorato, Juan Alcalde, Rafael Martinez-Monge, Natalia Zabalegui, Jesús Garcia-Foncillas

**Affiliations:** 1 Clinical Genetics Unit and Oncology Department, University Clinic of Navarra, Pamplona, Spain; 2 Otolaringology Department, University Clinic of Navarra, Pamplona, Spain; 3 Radiotherapy-Oncology Department, University Clinic of Navarra, Pamplona, Spain; 4 Immunotherapy Laboratory, CIMA University of Navarra, Pamplona, Spain

**Keywords:** real-time PCR, prognosis, molecular markers, head and neck cancer, TAK1

## Abstract

Resistance to radio and chemotherapy is one of the major drawbacks in the progression of head and neck squamous cell cancer (HNSCC) patients, evidencing the importance of finding optimum molecular prognosis markers to develop personalized treatment schedules. TGF-β effector TAK1 activity has been related to a greater aggressiveness in several types of cancer (Kondo et al. 1998; Edlund et al. 2003; Kaur et al. 2005) and, although there has been described no significant implication of TAK1 in HNSCC development, we have further examined the role of its mRNA expression as a marker of prognosis in HNSCC. Fifty-nine advanced HNSCC patients were recruited for the study. The tumor expression of TAK1 mRNA was analyzed with RT-PCR using Taqman technology and its relationship with the clinical outcome of the patients studied. TAK1 mRNA expression was lower in patients that relapsed than in those that did not, but the difference was only significant between the patients that showed response to treatment (p < 0.001). ROC curve analyses pointed a 0.5 expression ratio TAK1/B2M value as an optimum cut-off point for relapse and response. Our data suggest the TAK1 mRNA analysis by Taqman RT-PCR can predict the risk of relapse in HNSCC patients.

## Introduction

Cancers of the head and neck (HNSCC) account for 5% of all cancers detected per year. At least 90% are diagnosed as squamous cell carcinomas (Moore et al. 2004). Patients with locally advanced HNSCC enclose a bad prognostic group with less than 30% of surviving patients five years since finishing treatment, despite the improvements in surgery and new therapeutic approaches. Although clinical prognosis factors that determine tumor response to treatment have already been widely studied, less has been done in the field of the molecular factors with only several of them having a prognostic value defined by previous authors (Jayasurya et al. 2004; Smith et al. 2001).

TGF-β signalling pathway involvement in head and neck tumorogenesis through its growth inhibitory effect (Paterson et al. 2001; Prime et al. 2004; Lu et al. 2004; Rosenthal et al. 2004) has been widely studied (Prime et al. 2004; Paterson et al. 2001; Rosenthal et al. 2004; Garrigue-Antar et al. 1995; Lu et al. 2004; Muro-Cacho et al. 1999; Pasche, 2001). TGF-β-activated kinase 1 (TAK1) was originally identified as an MAP-3-kinase activated downstream of TGF-β/BMP receptors, positively regulating the SAPK/JNK and p38 kinase pathways (Yamaguchi et al. 1995). Since then, it has been identified as an effector in other signaling cascades aside TGF-β (Shim et al. 2005) such as, TNF (Srivastava et al. 2007), IL-1 (Choi et al. 2007) or lymphocyte differentiation (Shim et al. 2005; Delaney and Mlodzik, 2006). TAK1 activates the inhibition of cyclins D1 and A expression apart from mediating in BMP-2 inducible apoptosis (Wang et al. 1997). These pathways suppress cell growth in response to stress signals such as radiation and chemical damage (Singhirunnusorn et al. 2005; Wang et al. 1997). This role as tumor suppressor has been previously reported regarding lung cancer (Kondo et al. 1998), prostate cancer (Edlund et al. 2003) and liver cancer (Kaur et al. 2005). These studies established that the tumor tissue presents low levels of TAK1 expression or activity linked to a greater aggressiveness of disease, but there has not been described any significant implication of TAK1 in the development of HNSCC.

In the present study, we have further examined the role of TAK1 mRNA expression as a candidate to become a marker of prognosis in HNSCC.

## Materials and Methods

### Treatment

Fifty nine patients were recruited with AJCC stage II, III and IV after anatomopathological diagnosis of HNSCC (Table 1). They were enrolled in the study with approval from the institutional review board and after giving informed consent. Biopsies were collected at diagnosis and stored with RNA later^®^ (Ambion, Austin, TX) at −80 °C.

Treatment consisted of preoperative radiotherapy-acceleration fractionation with concomitant boost (72 Gy) and two cycles of concomitant cisplatin (20 mg/sqm/day, days 1–5 and 29–33, by continuous perfusion). Patients with a creatinine level above 1.3 mg/ml received carboplatin 60 mg/m^2^/day in the same time. All patients with normal cardiac function receive amifostine, 200 mg/m^2^ intravenously by 3 minutes infusion, previously to the first fraction of irradiation during the treatment course to prevent the toxic effects of the treatment. Surgical resection was carried out 3–4 weeks after the end of radiotherapy. After the preoperative treatment, patients were re-evaluated with fiberscopy and CT scanning following the RECIST guidelines (Husband et al. 2004). To proceed, patients were required to have adequate bone marrow recovery (defined as WBC count ≥ 100000cells/μl). After surgical resection, a pathological complete response (CR) was defined as the absence of residual tumor in the primary tumor and lymph nodes.

### Response assessment

To evaluate the clinical response to treatment WHO bidimensional criterion were followed (Therasse et al. 2000; Miller et al. 1981). To simplify the clinical data harvesting, stabilised and progressive disease were classified as “No Response”, therefore Complete and Partial response were classified as “Response”.

### RNA isolation and cDNA synthesis

Total RNA was isolated from tumor tissue using a Trizol^®^ reagent (Invitrogen, Breda, The Netherlands)/RNeasy mini columns (Qiagen, Valencia, CA, U.S.A.) hybrid protocol; 25 mg of the frozen tumoral tissue was dipped in 1.5 ml of Trizol^®^ reagent. Tissue samples were homogenized with Utra-Turrax homogenizer (Ika^®^-Werke, Schott Ibérica, S.A. Barcelona, Spain) and stored at room temperature (RT) for 5 minutes. 150 μl of chloroform 100% were added to the homogenate and shake vigorously for 15 seconds and then allowed to settle at RT for 10 minutes. After centrifugation at 12000 g 15 min at 4 °C the aqueous phase (top) was transferred to a new tube. Then, 375 μl of isopropanol 100% were added to each of the tubes and allow to sedimentate at room temperature for 10 minutes. The isolated RNA was precipitated by centrifugation at 12000 g 10 minutes at 4 °C and washed with 1 ml of ethanol 75% and further spin at 7500 g during 10 minutes. The RNA was dried at room temperature and underwent subsequent wash and DNase treatment with the Quiagen RNeasy columns according to the manufacturer’s protocol. RNA quantity was measured with the NanoDrop^®^ ND-1000 UV-Vis Spectrophotometer (NanoDrop Technologies), and its quality was tested through 2% agarose gel electrophoresis.

To generate complementary DNA (cDNA), total RNA was reverse transcribed using 2.5 units/μl of murine leukemia virus reverse transcriptase, 2.5 μM random hexamers, dNTPs (2 mM each) and 1 unit/μl of RNase inhibitor (Applied Biosystems, Rotkreuz, Switzerland). The reverse transcriptase reaction was performed in a total volume of 100 μl in a GeneAmp polymerase chain reaction cycler (Applied Biosystems, Rotkreuz, Switzerland) at 25 °C for 10 minutes, followed by 30 minutes at 48 °C and 5 minutes at 95 °C. The cDNA was stored at −20 °C until its further analysis.

### Housekeeping gene

In order to choose the most appropriate housekeeping gene for the real-time PCR analysis, the assay “TaqMan^®^ Human Endogenous Control Plate” (Applied Biosystems, Rotkreuz, Switzerland) was performed with three randomly chosen samples. The most suitable housekeeping gene was β-2-microglobulin (B2M) (data not shown).

### Real time-PCR assays

Real Time-PCR analyses were performed with the commercial assays “Assay on the Demand” from Applied Biosystems (Rotkreuz, Switzerland). Assay number Hs00177373_m1 was employed for the TAK1 mRNA and assay number Hs99999907_m1 for the housekeeping gene B2M mRNA.

PCR amplifications were performed on ABI Prism 7700 SDS (Applied Biosystems, Rotkreuz, Switzerland). The reactions contained 12.5 μl of TaqMan Universal Master Mix (which includes amplitaq gold DNA polymerase, dNTP’s with UTP, MgCl_2_, ROX, Amperase UNG and buffers), 1.25 μl of each commercial *Assay on Demand* and 5 μl of cDNA template. Reactions were carried out in ABI Prism 96-well optical reaction plates with 96-well optical covers (Applied Biosystems, Rotkreuz, Switzerland) under Universal Cycling Standard Conditions (2 min at 50 °C, 10 min at 95 °C and 40 cycles of 15 s at 95 °C, 1 min at 60 °C). Each sample was analyzed three times to obtain two replicates of the measure.

### Standard curve and amplification efficiency

A control RNA (Control Human RNA, Applied Biosystems, Rotkreuz, Switzerland) was included in the study as quality control and as reference to perform the expression differences calculations. With this RNA a standard curve for each gene, including the housekeeping gene, was added in each assay. The curves were done in order to provide the efficiency of each reaction (E% = 10^−1/slope^−1)*100), and to deduce the curve equation that allowed to calculate the relative dilution of each gene in each sample. This is a way to improve the measurements by normalizing not only by the housekeeping gene expression but also by the expression levels in the control sample.

### Relative expression calculation methods

**Figure 1:**
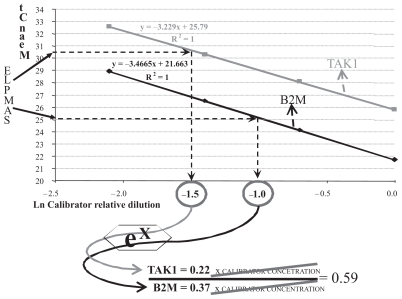
Using the control RNA as a “Calibrator Sample”, we built two standard curves or lines, one for the housekeeping gene, B2M, and one for the gene in study, TAK1. These standards enabled the control of the efficiency of the reaction in each experiment performed and, at the same time, they served as a third level of normalization for the data obtained from Real Time PCR. In particular, we used them to calculate in each sample the concentration of each mRNA, the housekeeping gene’s and TAK1 gene’s relative to the concentration of these mRNAs in the Calibrator sample. Calculating the ratio between these two Relative Concentrations we obtained a dimensionless measure directly proportional to the TAK1 mRNA amount expressed in each tissue sample tested. In the example exposed in the figure we calculated for a sample a relative expression value of TAK1 of 0.59 extrapolating the mean Ct values obtained in the Real Time PCR measurement in the standard curves/lines built with consecutive dilutions of the Calibrator mRNA. Once obtained a value of Ln Calibrator relative dilution for each gene, we raised e to that value and calculate the dimensionless ratio we were searching.

The equation used to calculate the expression ratio for each gene in each sample was: *Ratio Gene X = Relative Dilution gene X/Relative Dilution B2M.* The obtained data was a relative ratio normalized firstly against the sample housekeeping gene expression. Following, it was again normalized by using the expression of the housekeeping gene and the gene in study (TAK1) in the control mRNA (Fig. 1).

### Statistical analysis

SPSS 11.0 software was employed to perform statistical analysis. Expression ratio values were transformed by logarithm in order to test the normality distribution. The normality status was checked with the Shapiro-Wilk test. The expression of TAK1 was compared among the different clinical and pathological variable and response patterns by using one-way ANOVA tests and the post-hoc tests LSD or Tanhame depending of the homogeneity of variance.

The accuracy of the TAK1 expression levels measurements in the response to treatment prediction was assessed on the basis of the area under the ROC curve (*Receiver Operating Characteristic*). The optimal ROC curve threshold matched the size breakpoint showing improved sensitivity and specificity. The area under the curve 0.7 to 0.9 value indicated moderate accuracy. In addition, the sensitivity, specificity, and positive and negative predictive values were calculated for several cutoff points obtained from the ROC curve. To assess the differential distribution of the patients according to the ROC designated cutpoint a Chi Square test was performed.

## Results

### RT-PCR expression measurements

The calculation method applied in this study reports the expression levels as non-dimensional concentration rates. These concentrations belong to the TAK1 gene mRNA and the housekeeping gene B2M mRNA analyzed in each sample. The control sample allowed building two separate standard lines for the target gene, TAK1, and the housekeeping gene B2M. Standard lines that are employed to extrapolate the results obtained for the target gene mRNA and the housekeeping gene mRNA. The data obtained are relative concentrations to the control sample and not real concentrations. Therefore the final ratio expresses, in a directly proportional way, the target gene mRNA levels that each sample contains.

However as the variable “TAK1/B2M expression ratio” did not follow a normal distribution, each value was transformed with neperian logarithm. The transformed variable fitted a normal distribution (P_Kolmogorov-Smirnov_ >0.05).

### Clinical outcome

The clinical data obtained at the end of the following period (Table 2) showed that none of the patients included in the study achieved a complete response to treatment.

The majority of the patients did not develop severe toxicity to radiotherapy, nine patients suffered distant metastasis, most of them located in the lungs and within fourteen months since the end of the treatment. But almost half of the studied patients (45%) suffered from local relapses of the disease and the median survival without them was of twenty-four and a half months.

The statistical analysis showed no significant relationship among the different clinical variables considered including clinical data such as tobacco consumption or gender (Table 1).

### TAK1 mRNA expression levels, response to treatment and local relapse development

The studied patients showed a different expression pattern depending on the achieved response to the applied treatment. The interaction analysis showed that this differential pattern is modulated by the local relapse development (p = 0.02).

According to the statistical analysis, patients that developed response to treatment showed higher tumoral TAK1 mRNA expression levels than those that did not achieve any response. At the same time this expression levels were also elevated in the tumor tissue of the patients that did not develope a local relapse in contrast to those that suffered a local relapse.

**Figure 2:**
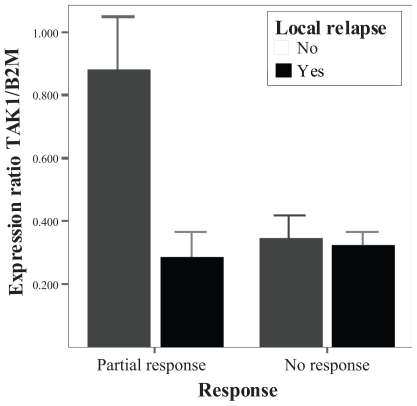
Contrast analysis of the interaction RESPONSE-RELAPSE regarding TAK1 mRNA tumor expression. In the columns situated in the left of the graphic, it can be observed that the expression levels difference, between those patients that suffered relapse and those that did not, is statistically significant among the patients that achieved some response to treatment.

Among patients that showed response to treatment, those that developed a local disease relapse despite the treatment efficiency had lower tumor levels of TAK1 mRNA than those that did not relapse (p < 0.001). At the same time, when the data were analyzed among patients that suffered a local relapse, patients that achieved response to treatment had higher tumoral levels of TAK1 mRNA than the patients that did not respond to treatment (p < 0.01). (Fig. 2)

**Figure 3:**
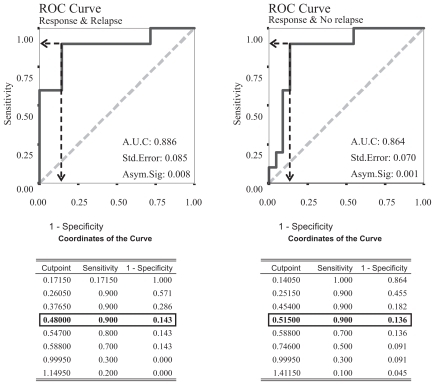
ROC analysis for the previous contrast results. The curve on the left hand side analyzes the tumour TAK1 mRNA expression from the patients with response to treatment searching for a cut point for the presence of relapse; the second curve (right side) analyzes the differences in the patients that although relapsed locally showed some response to treatment and those that did not respond. Both curves present a high exactitude (Area Under the Curve approximately 0.9) and both curves point to the same TAK1 mRNA expression level in the tumour tissue as a cut off point between bad and good prognosis in these patients: a TAK1/B2M expression ratio of 0.5.

As the expression differences were highly significant ROC curve (*Receiver Operating Characteristic curve*) analysis was applied searching for an expression level as cut-off point for response to treatment and relapse (Fig. 3).

Both analyzed ROC curves showed similar points with maximum specificity and sensitivity. Both pointed that a 0.5 value of expression ratio TAK1/B2M as an optimum cut-off point for relapse and respond.

**Figure 4:**
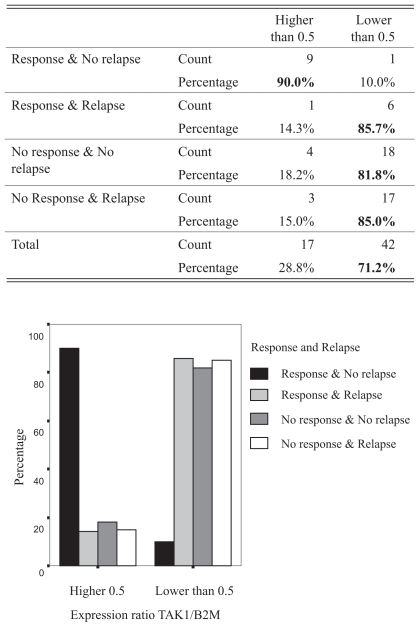
The table shows the patients’ distribution depending on their TAK1/B2M expression ratio related to the ROC curves’ designated cut off point of 0.5. The graphic represents the patient’s distribution according to their response pattern, relapse development pattern and the TAK1/B2M expression ratio. It can be observed that the former ROC curves have successfully found a TAK1 expression ratio that discriminates patients according to their clinical outcome, 90.0% of the ones with better prognosis, no relapse and response, having TAK1 mRNA expression ratios over 0.5, whereas most of the patients that present relapse, no response to treatment or both express TAK1 mRNA in the tumour tissue at lower levels than this designated ROC curve cut off point.

Chi square analysis of the relationship between response and relapse and a TAK1/B2M expression ratio lower or higher than 0.5 revealed that the vast majority of the patients that developed response to treatment and did not suffer a local disease relapse had an expression ratio higher than 0.5 (90.0%), while the majority of patients that did not respond to treatment or, despite developing some response to the treatment, suffered a local relapse had TAK1/B2M expression ratio levels lower than 0.5 (response and relapse 85.7%, no response but no relapse 81.8% and relapse and no response 71.2%) (p< 0.05). (Fig. 4)

## Discussion

Growth regulatory proteins of the transforming growth factor-β family (TGF-β1) are one of the few classes of endogenous inhibitors of cell growth. These cytokines have been implicated in diverse phenomena including growth control, cell adhesion and motility, production of extracellular matrix components, and alteration of cell phenotype (Massague, 1998). An important question is how cancer cells escape from normal growth regulatory mechanisms to become malignant, and further, which events favour progression and metastasis. Therefore the comprehension of the roles played by the implicated molecular factors in that control deregulation is essential. At the same time, translation of these data to the clinical environment can improve not just the development of new therapeutical systems, but also the prognosis and subsequent treatment schedule of cancer patients.

There are many studies in different types of human tumour that have shown that altered inhibition of TGF-β1 cell proliferation during malignant transformation is due to down-regulation of the TGF-β1 receptor system (Derynck et al. 2001; Hata et al. 1998; Paterson et al. 2001; Yamada et al. 2001; Edlund et al. 2003; Lee et al. 2001; Pasche, 2001). Although the most studied downstream pathway of the TGF-β system are the SMAD pathways, it has been demonstrated, in cell lines, that the specific activation of the p38 MAP kinase pathway is essential for TGF-β1-induced apoptosis (Edlund et al. 2003). The main TGF-β activated kinase in this pathway is TAK1 (Yamaguchi et al. 1995; Wang et al. 1997). Kaur et al. (2005) described that hepatic cancer cell lines refractory to the TGF-β antitumoral properties have lower levels of TAK1 activity (Kaur et al. 2005). Edlund et al. (2003) found that negative TAK1 prostate cancer cell lines had reduced TGF-beta1-induced phosphorylation of p38 and apoptosis, and described how endogenous SMAD7 interacts with TAK1 acting as a scaffolding protein in the regulation of p38 (Edlund et al. 2003). Although TAK1 participates not only in TGF-β signalling but also in many other molecular pathways (Delaney and Mlodzik, 2006). It responds to a variety of upstream signals, including inflammatory molecules and developmental cues, therefore acting as a common effector in regulating cellular responses to stress signals coming from different molecular sensors.

Based on these findings and the described signalling mechanism of TAK1 with its downstream targets we suggest that TAK1 downregulation can be also implicated in HNSCC genesis and clinical development. We have found not just that the patients studied showed a relationship between reduced TAK1 tumor mRNA expression and poor clinical prognosis, but also that a predictive cutoff value can be set. Therefore TAK1 mRNA expression could be used as a prognosis predictive marker in HNSCC patients. In our study a relative expression ratio value lower than 0.5 would distinguish patients with poor response and clinical prognosis.

The application of RT-PCR to the clinical environment can provide an accurate and fast tool to evaluate certain markers’ levels, being one of them the TAK1 mRNA tumor levels in HNSCC as the present works establishes. Messenger RNA levels may not provide information about the protein activity, but it does provide information about the tumor cell status. An extremely valuable information as the average survival rate of advanced Head and Neck patients has hardly been increased in the past ten years despite the improvements in treatment schedules (Hernández, 2000).

This study, therefore, encourages further investigation in the mechanisms that regulate TAK1 in the particular case of HNSCC. Once established that deregulation of the TGF-β signalling pathway is implicated in head and neck tumorogenesis (Prime et al. 2004; Derynck et al. 2001; Paterson et al. 2001; Rosenthal et al. 2004; Wang et al. 1997; Garrigue-Antar et al. 1995; Lu et al. 2004; Muro-Cacho et al. 1999; Pasche, 2001), the particular mechanisms which TAK1 employs to modulate the tumor cell behaviour in the HNSCC case have to be uncovered. Our results suggest that Head and Neck tumor cells may overcome the pro-apoptotic effects of TGF-β signalling pathways through a downregulation of TAK1 activity, and that one mechanism of downregulation of this activity can be the inhibition of TAK1 mRNA synthesis as we have found its levels decreased.

## Figures and Tables

**Table 1:** Basic clinical data of the patients included in the study. Including risk factor tobacco, age of onset, gender, and histological data of the tumour tissue biopsied at onset.

	Patients	Total%
**Gender**	**59**	**100**
Male	49	83.1
Female	10	16.9
**Diagnosis medium age (years)**		58.9
**Tobacco**		
Yes	44	74.6
No	15	25.4
**Daily tobacco**		
One packet	34	77.3
Two packets	10	22.7
**AJCC**		
II	5	8.5
III	14	23.7
IV	40	67.8
**Histologic differentiation grade**		
Well	13	22.0
Moderate	37	62.7
Poor	9	15.3

**Table 2:** Clinical data of the patients included in the study at the end of the following period. The Final clinical status is codified as: alive without disease (AWOD), alive with disease (AWD), dead without disease (DWOD) and dead with disease (DWD). Relapse and metastasis free survival medians are expressed in months.

	Patients	Total%
**Response**	**59**	**100**
Complete response	0	0
Partial response	17	28.8
Stable disease	42	71.2
**Toxicity to radiotherapy**		
No toxicity	26	44.1
II	20	33.9
III	11	18.6
IV	2	3.4
**Final clinical status**		
AWOD	29	49.2
AWD	15	25.4
DWOD	8	13.6
DWD	7	11.9
**Local relapse development**		
No	32	54.2
Yes	27	45.8
**Metastasis development**		
No	50	84.7
Yes	9	15.3
**Metastasis localization**		
No metastasis	50	84.7
Lung	6	10.2
Bone	1	1.7
Lymph node	5	8.5
Liver	4	6.8
Skin	2	3.4
**Median relapse free survival (months)**		24.5
**Median metastasis free survival (months)**		14.0

## Abbreviations

HNSCC: Head and Neck Squamous Cell Cancer
RT-PCR: Real Time-Polymerase Chain Reaction
ROC: Receiver Operating Characteristic
AJCC: American Joint Committee on Cancer
RECIST: Response Evaluation Criteria in Solid Tumors
CR: Complete Response to Treatment
PR: Partial Response to Treatment
NR: No Response to Treatment
WHO: World Health Organization
RT: Room Temperature
cDNA: Complementary DNA
mRNA: Messenger RNA
P: Statistical Probability
NCBI: National Center for Biotechnology Information

## References

[b1-grsb-2008-063] Choi K Lee J Choi C 2007 FEBS Lett 581 4691 6 17854800 10.1016/j.febslet.2007.08.065

[b2-grsb-2008-063] Delaney JR Mlodzik M 2006 Cell. Cycle 5 2852 5 17218788 10.4161/cc.5.24.3558

[b3-grsb-2008-063] Derynck R Akhurst RJ Balmain A 2001 Nat. Genet 29 117 29 11586292 10.1038/ng1001-117

[b4-grsb-2008-063] Edlund S Bu S Schuster N Aspenstrom P Heuchel R Heldin NE ten Dijke P Heldin CH Landstrom M 2003 Mol. Biol. Cell 14 529 44 12589052 10.1091/mbc.02-03-0037 PMC149990

[b5-grsb-2008-063] Garrigue-Antar L Munoz-Antonia T Antonia SJ Gesmonde J Vellucci VF Reiss M 1995 Cancer Res 55 3982 7 7664267

[b6-grsb-2008-063] Hata A Shi Y Massague J 1998 Mol. Med. Today 4 257 62 9679244 10.1016/s1357-4310(98)01247-7

[b7-grsb-2008-063] Hernández JJC 2000 Cáncer de Cabeza y Cuello GRUPO Aula Médica S.A., Madrid

[b8-grsb-2008-063] Husband JE Schwartz LH Spencer J Ollivier L King DM Johnson R Reznek R 2004 Br. J. Cancer 90 2256 60 15150551 10.1038/sj.bjc.6601843 PMC2410289

[b9-grsb-2008-063] Jayasurya R Francis G Kannan S Lekshminarayanan K Nalinakumari KR Abraham T Abraham EK Nair MK 2004 Int. J. Cancer 109 710 6 14999779 10.1002/ijc.20042

[b10-grsb-2008-063] Kaur S Wang F Venkatraman M Arsura M 2005 J. Biol. Chem 280 38599 608 16157589 10.1074/jbc.M505671200

[b11-grsb-2008-063] Kondo M Osada H Uchida K Yanagisawa K Masuda A Takagi K Takahashi T 1998 Int. J. Cancer 75 559 63 9466656 10.1002/(sici)1097-0215(19980209)75:4<559::aid-ijc11>3.0.co;2-4

[b12-grsb-2008-063] Lee S Cho YS Shim C Kim J Choi J Oh S Zhang W Lee J 2001 Int. J. Cancer 94 500 7 11745435 10.1002/ijc.1494

[b13-grsb-2008-063] Lu SL Reh D Li AG Woods J Corless CL Kulesz-Martin M Wang XJ 2004 Cancer Res 64 4405 10 15231647 10.1158/0008-5472.CAN-04-1032

[b14-grsb-2008-063] Massague J 1998 Annu. Rev. Biochem 67 753 91 9759503 10.1146/annurev.biochem.67.1.753

[b15-grsb-2008-063] Miller AB Hoogstraten B Staquet M Winkler A 1981 Cancer 47 207 14 7459811 10.1002/1097-0142(19810101)47:1<207::aid-cncr2820470134>3.0.co;2-6

[b16-grsb-2008-063] Moore RJ Chamberlain RM Khuri FR 2004 Support Care Cancer 12 338 46 15064931 10.1007/s00520-003-0532-y

[b17-grsb-2008-063] Muro-Cacho CA Anderson M Cordero J Munoz-Antonia T 1999 Clin. Cancer Res 5 1243 8 10389906

[b18-grsb-2008-063] Pasche B 2001 J. Cell. Physiol 186 153 68 11169452 10.1002/1097-4652(200002)186:2<153::AID-JCP1016>3.0.CO;2-J

[b19-grsb-2008-063] Paterson IC Matthews JB Huntley S Robinson CM Fahey M Parkinson EK Prime SS 2001 J. Pathol 193 458 67 11276004 10.1002/1096-9896(2000)9999:9999<::AID-PATH822>3.0.CO;2-V

[b20-grsb-2008-063] Prime SS Davies M Pring M Paterson IC 2004 Crit. Rev. Oral Biol. Med 15 337 47 15574678 10.1177/154411130401500603

[b21-grsb-2008-063] Rosenthal E McCrory A Talbert M Young G Murphy-Ullrich J Gladson C 2004 Mol. Carcinog 40 116 21 15170816 10.1002/mc.20024

[b22-grsb-2008-063] Shim JH Xiao C Paschal AE Bailey ST Rao P Hayden MS Lee KY Bussey C Steckel M Tanaka N Yamada G Akira S Matsumoto K Ghosh S 2005 Genes Dev 19 2668 81 16260493 10.1101/gad.1360605 PMC1283960

[b23-grsb-2008-063] Singhirunnusorn P Suzuki S Kawasaki N Saiki I Sakurai H 2005 J. Biol. Chem 280 7359 68 15590691 10.1074/jbc.M407537200

[b24-grsb-2008-063] Smith BD Haffty BG Sasaki CT 2001 Ann. Otol. Rhinol. Laryngol 110 221 8 11269765 10.1177/000348940111000304

[b25-grsb-2008-063] Srivastava AK Qin X Wedhas N Arnush M Linkhart TA Chadwick RB Kumar A 2007 J Biol Chem 10.1074/jbc.M705329200 PMC4154379 17897957

[b26-grsb-2008-063] Therasse P Arbuck SG Eisenhauer EA Wanders J Kaplan RS Rubinstein L Verweij J Van Glabbeke M van Oosterom AT Christian MC Gwyther SG 2000 J. Natl. Cancer Inst 92 205 16 10655437 10.1093/jnci/92.3.205

[b27-grsb-2008-063] Wang W Zhou G Hu MC Yao Z Tan TH 1997 J. Biol. Chem 272 22771 5 9278437 10.1074/jbc.272.36.22771

[b28-grsb-2008-063] Yamada H Vijayachandra K Penner C Glick A 2001 J. Biol. Chem 276 19052 8 11262404 10.1074/jbc.M100615200

[b29-grsb-2008-063] Yamaguchi K Shirakabe K Shibuya H Irie K Oishi I Ueno N Taniguchi T Nishida E Matsumoto K 1995 Science 270 2008 11 8533096 10.1126/science.270.5244.2008

